# Microbial metabolomics in open microscale platforms

**DOI:** 10.1038/ncomms10610

**Published:** 2016-02-04

**Authors:** Layla J. Barkal, Ashleigh B. Theberge, Chun-Jun Guo, Joe Spraker, Lucas Rappert, Jean Berthier, Kenneth A. Brakke, Clay C. C. Wang, David J. Beebe, Nancy P. Keller, Erwin Berthier

**Affiliations:** 1 Department of Biomedical Engineering, University of Wisconsin-Madison, Madison, Wisconsin 53705, USA; 2 Carbone Cancer Center, University of Wisconsin-Madison, Madison, Wisconsin 53705, USA; 3 Department of Urology, University of Wisconsin-Madison, Madison, Wisconsin 53705, USA; 4 Department of Pharmacology and Pharmaceutical Sciences, University of Southern California, Los Angeles California 90089, USA; 5 Department of Plant Pathology, University of Wisconsin-Madison, Madison, Wisconsin 53705, USA; 6 Department of Medical Microbiology and Immunology, University of Wisconsin-Madison, Madison, Wisconsin 53705, USA; 7 Department of Biotechnology, CEA-University Grenoble-Alpes, 17 Avenue des Martyrs, 38054 Grenoble, France; 8 Department of Mathematics, Susquehanna University, Selinsgrove, Pennsylvania 17870, USA; 9 Department of Chemistry, University of Southern California, Los Angeles California 90089, USA; 10 Department of Bacteriology, University of Wisconsin-Madison, Madison, Wisconsin 53705, USA

## Abstract

The microbial secondary metabolome encompasses great synthetic diversity, empowering microbes to tune their chemical responses to changing microenvironments. Traditional metabolomics methods are ill-equipped to probe a wide variety of environments or environmental dynamics. Here we introduce a class of microscale culture platforms to analyse chemical diversity of fungal and bacterial secondary metabolomes. By leveraging stable biphasic interfaces to integrate microculture with small molecule isolation via liquid–liquid extraction, we enable metabolomics-scale analysis using mass spectrometry. This platform facilitates exploration of culture microenvironments (including rare media typically inaccessible using established methods), unusual organic solvents for metabolite isolation and microbial mutants. Utilizing *Aspergillus*, a fungal genus known for its rich secondary metabolism, we characterize the effects of culture geometry and growth matrix on secondary metabolism, highlighting the potential use of microscale systems to unlock unknown or cryptic secondary metabolites for natural products discovery. Finally, we demonstrate the potential for this class of microfluidic systems to study interkingdom communication between fungi and bacteria.

## Abstract (web summary)

Traditional methods for microbial culture and subsequent metabolomics are time-consuming and labour-intensive. Here the authors present a microscale culture platform with integrated extraction for efficient, low-volume metabolomics of relevant microenvironments and microbial co-cultures.

## Graphical abstract

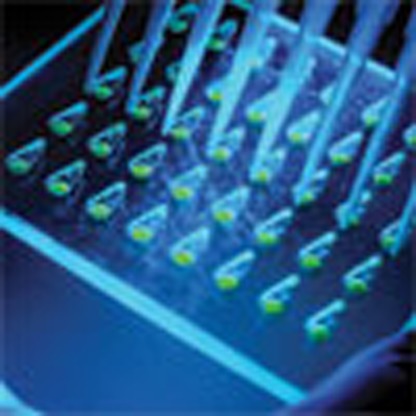

Microbial secondary metabolism is an incredibly complex source of bioactive compounds that have important implications for human, animal and plant health. Filamentous fungi, in particular, produce secondary metabolites that are key virulence determinants of human and plant disease^1^,^2^, a prominent threat to food and feed supplies^3^,^4^, and a rich source of therapeutic compounds^5^. As only a small fraction of the potential fungal metabolite pool has been discovered, the identification of novel fungal compounds is the focus of much current interest^6^,^7^. However, studying fungal secondary metabolites is particularly challenging because they are often produced in response to very specific environmental cues (temperature, available nutrients, signals from nearby organisms) to provide a competitive advantage to the fungus^8^,^9^,^10^. Interactions with adjacent organisms, including bacteria and insects (so-called multikingdom interactions), can also have a significant impact on fungal secondary metabolite production^9^,^10^. Indeed, genome sequencing of hundreds of fungi has found innumerable cryptic secondary metabolite clusters not expressed under traditional laboratory growth conditions^11^,^12^,^13^. Identification of the environmental inducers of these clusters is, however, limited by current fungal culture and metabolite isolation tools that do not allow a simple and time-efficient exploration of the wide range of culture conditions representative of those found in nature.

Traditional methods of fungal culture use flasks or petri dishes, which require large volumes of reagents, incubator or shaker space and significant processing time. An important and time-consuming step in metabolomic analysis is sample preparation to remove matrix effects from culture media (such as salts, proteins and cell debris) that could impact downstream liquid chromatography-mass spectrometry (LC-MS) results. This is most commonly achieved by either solid-phase or liquid–liquid extraction, but these processes are time consuming and manually intensive as solid cultures must first be homogenized while liquid cultures are typically centrifuged or filtered. Liquid–liquid extraction is also imprecise as the immiscible phase extraction step is a highly serial process, with each pipetting step requiring precise selection of the location of the fluid interface. As such, there is a need for a culture method that has a small physical footprint, is efficient to use and is compatible with a passive, reproducible small molecule extraction process.

Here we address these obstacles by presenting a microscale platform that simplifies and accelerates the workflow of secondary metabolism studies, allows the exploration of a larger spectrum of microenvironmental cues, and brings salient features of microscale platforms (surface/volume ratios, segregated culture chambers, matrix design) to bear on microbial metabolomics research. We leverage microfluidic interfaces^14^ to create an open biphasic system in which organic solvent is guided over a microbial culture environment allowing for the integrated and passive extraction of metabolites. These methods build on advances made in open and suspended microfluidics (microfluidics in channels that have any number of open interfaces) that have demonstrated unique advantages for mammalian cell culture and metabolomics^15^. The open microscale culture and extraction technologies presented here are intended to demonstrate simple microbial culture and extraction concepts that can integrate with many other microfluidic methods that provide enhancements in the concentration of secreted factors, the use of rare samples, and the creation of physiologically relevant *in vitro* models^16^,^17^,^18^. We used these devices to perform multidimensional arrayed experiments, varying culture conditions and metabolite isolation conditions, and demonstrate that efficiency of extraction from microscale is sufficient for LC-MS analysis. Using fungi well known for their rich secondary metabolism, *Aspergillus nidulans* (*A. nidulans*) and *Aspergillus fumigatus* (*A. fumigatus*), as model systems, we demonstrate the potential of a combinatorial approach to metabolite extraction and microbial culture for microbial metabolomics. Finally, we designed an integrated coculture and extraction platform that is capable of performing cultures of fungi and bacteria and further enables studies of chemical interactions between kingdoms.

## Results and discussion

### Micrometabolomics platform

**Figure 1:**
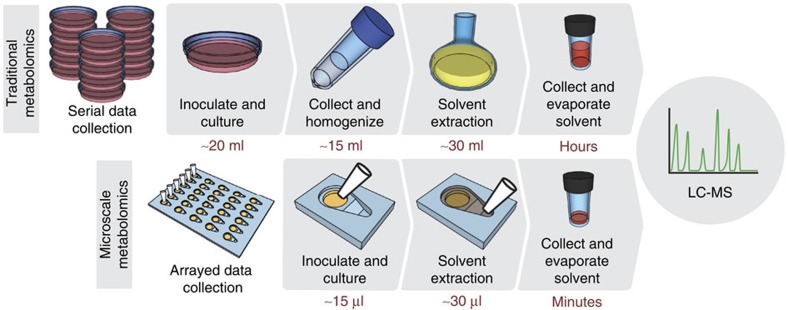
The micrometabolomics platform workflow is simpler, faster, and takes up less space than traditional metabolite extraction. Traditional fungal metabolomics workflows (top) require serial inoculation of cultures, collection and homogenization of the sample, extraction of metabolites with solvent, and finally evaporation of that solvent prior to analysis. The microscale workflow (bottom) allows for arrayed inoculation and on-chip metabolite extraction without the need for culture collection and homogenization. Besides the streamlined process, the micrometabolomics platform also uses ∼1000 × less solvent, which cuts down on evaporation time and makes the workflow faster.

**Figure 2:**
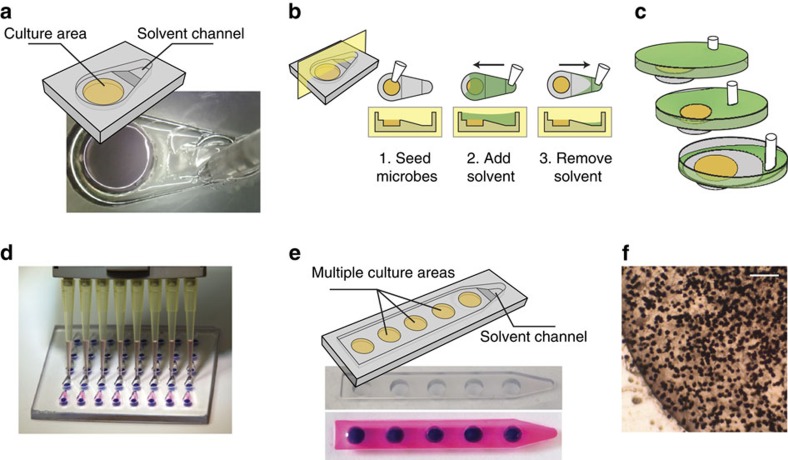
Extraction at microscale. (**a**) The micrometabolomics device is comprised of a central culture well with an overlying pipet-accessible solvent channel. (**b**) The device is operated in three simple steps. (**c**) Simulations of fluid flow in the platform demonstrate that solvent removal does not disturb the liquid culture underneath. (**d**) The devices are arrayable and compatible with a multichannel pipette. (**e**) The extraction module can also be integrated with a platform for pooled extraction. (**f**) Sporulating culture of *A. fumigatus* overlayed on solid GMM agar grown in the micrometabolomics platform for 2 days. Scale bar, 250 μm.

We engineered a microscale metabolomics platform that satisfies requirements of microbial culture and solvent flow (known as spontaneous capillary flow^15^,^19^) in open microfluidic channels. Using this platform, we demonstrate the concept of microscale microbial metabolomics (Fig. 1). In the micrometabolomics device, a micro-agar pad or liquid well is used to culture the microorganisms within an open microfluidic channel (for example, using a teardrop-shaped channel as exemplified in Fig. 2a). Metabolite extraction is simple to perform with a pipette; the open microfluidic channel is designed to direct the flow of solvent over the aqueous culture areas and form stable biphasic interfaces (Fig. 2b). This surface tension-based stability of the aqueous component is confirmed by numerical modelling using the Evolver software^20^ (Fig. 2c; Supplementary Fig. 1), as well as experimentally (Supplementary Movie), provided the solvent is less dense than the aqueous media. An important factor contributing to the stability is the radially symmetrical covering of the aqueous interface. To facilitate symmetrical covering, we designed a device that drives filaments of solvent around the aqueous well before the solvent–aqueous interface is initiated (Supplementary Fig. 1A); this improves the evenness of solvent covering and prevents destabilizing perturbations. Once formed, the biphasic interface enables the extraction of metabolites based on preferential partitioning into the organic phase (Fig. 2b). At the end of the extraction, the solvent is collected in a second simple pipetting step; because the location of the liquid–solvent interface is precisely controlled, this step is repeatable, arrayable, automatable and does not require an additional processing step (for example, centrifugation). Importantly, the open microfluidic design allows the retrieval of solvent without carrying any aqueous media with it—an essential condition for sample preparation. The stability and integrity of the aqueous fluid was also validated by numerical simulation using the Evolver software (Fig. 2c; Supplementary Fig. 1B), which shows that during solvent retrieval, the solvent level decreases and the aqueous compartment becomes domed until the solvent breaks around an eye of aqueous media which grows in diameter eventually allowing the media to settle back in its original configuration. The solvent is then evaporated and the metabolites are analysed by LC-MS. The efficiency of extraction in the micrometabolomics platform is comparable to traditional extraction techniques such as vortexing or homogenization (Supplementary Fig. 2).

The open nature of the micrometabolomics platform is especially enabling for fungal and bacterial cultures. Biologically, the open surface creates an air interface that conditions the sporulation of certain fungi and the production of specific secondary metabolites^21^,^22^. *A. fumigatus* grown in the device at the air interface retained its expected morphology (Fig. 2f). In addition, the open design avoids the external pumping methods common among most other microfluidic devices that would make screening experiments nearly impossible and fabrication challenging, effectively preventing widespread integration into biology labs. The concepts of open micrometabolomic methods developed in this work represent the simplest form of open-culture devices (Fig. 2a) and have been used to demonstrate the potential of microscale devices for combined culture and metabolomic analysis. These techniques can also be extended to more complex segregated cocultures (see below) or devices for pooled extraction (Fig. 2e) that would allow for an efficient screening strategy or analyses that require larger amounts of metabolites extracted while maintaining the microscale culture geometries.

The design of the micrometabolomics platform is particularly useful for experimental spaces involving many microenvironmental conditions, time points and strains. The micrometabolomics platform benefits from the small size of each device (Supplementary Fig. 3A; Supplementary Data 1), which makes it possible to array devices for a simpler, more systematic workflow. A set of 30 devices fits easily into the footprint of a double-width microscope slide (Fig. 2d), whereas a stack of 30 petri dishes occupies a shelf of an incubator. Perhaps more important is that the open platform is fast and easy to use; traditional methods of fungal secondary metabolite extraction require samples to be processed individually and use large volumes of solvent, both of which lead to long processing times. In contrast, the micrometabolomics platform processes samples in parallel using a simple micropipette (Fig. 2d), eliminates the homogenization step, and uses ∼1,000 × less solvent for the extraction, all of which contribute to a much faster workflow (Fig. 1). Even with the small culture and extraction volumes, the micrometabolomics platform recovers enough material to be compatible with LC-MS analysis.

Finally, the open microfluidic design confers significant advantages during fabrication. Traditional microfluidic platforms are commonly fabricated from materials that are incompatible with many solvents and known to sequester hydrophobic small molecules^23^,^24^. Open designs allow the use of a wider range of fabrication techniques (for example, injection molding) and of materials as bonding is not required^25^. Furthermore, by remaining completely open on top, the micrometabolomics device can be treated using deposition techniques, such as coating with Parylene C, to render the device material solvent resistant^26^. When similar deposition coating is performed on closed microfluidics, the treated surface is uneven throughout the device^27^.

### Metabolite profile depends on extraction solvent

**Figure 3:**
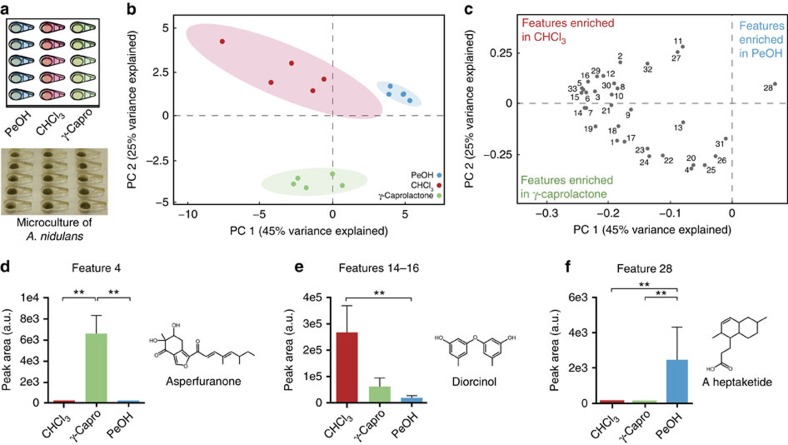
Solvent selection impacts extraction of secondary metabolites. (**a**) Experimental design and culture photo. PeOH is 1-pentanol, CHCl_3_ is chloroform and γ-Capro is γ-caprolactone. (**b**) Principal component analysis of *A. nidulans* cultured in the micrometabolomics platform. Only features that could be annotated as known secondary metabolites were used for clustering (Table 1). Each dot represents one of five independent cultures per condition from one experiment and the shaded ellipses represent 95% confidence intervals. Variance explained refers to the amount of total variation observed between samples that can be attributed to segregation along that principal component. (**c**) Loadings plot of individual features for the PCA in **b**. (**d**–**f**) Peak areas (integrated peak intensities, arbitrary units (a.u.)) of three of the features numbered in **c**. Error bars represent s.d. of the five replicates and statistics were performed using the Kruskal–Wallis test as described in the methods; ^**^P value <0.01. Peak areas for features 14–16 were summed as they are adducts of the same compound. Structures are of the putative annotation for each peak.

The ability to use a large range of solvents for the extraction process allows the study of diverse chemical structures^28^,^29^,^30^. To demonstrate the potential of our open microscale extraction system, we tested how varying the extraction solvent affected metabolite profiling from fungal cultures. We cultured *A. nidulans*—a fungal species with ∼40% of its secondary metabolome characterized^31^—on glucose minimal media (GMM)^32^ both in the micrometabolomics platform and at macroscale (conventional petri dish culture with flask-based extraction). We then extracted metabolites using three different solvents, chloroform, 1-pentanol, and γ-caprolactone, which were chosen to cover a range of polarities (Fig. 3a; Supplementary Table 1). As solvent volatility is less important in the microscale platform, we were able to use high boiling point solvents, pentanol (bp=138 °C) and γ-caprolactone (bp=219 °C), not typically employed in fungal secondary metabolite extraction. The chromatograms of microscale cultures extracted with the different solvents had visible differences (Supplementary Fig. 4), and to get at these changes, features extracted from the chromatograms were compared using principal component analysis (PCA). As untargeted metabolomics typically yields a large number of unidentifiable features, we first narrowed the comparison: only features that could be putatively annotated as secondary metabolites based on exact mass (error <10 p.p.m.) when compared with databases of known *A. nidulans* compounds were used in the PCA (Table 1; Fig. 3b). Of the ∼1,000 features observed by LC-MS, 33 features could be putatively annotated based on databases of known *A. nidulans* compounds. These 33 features correspond to 19 putative metabolites; as is commonly observed in metabolomics data, a single metabolite may form multiple adducts (for example, features 14–16 were annotated as three different diorcinol adducts). When considering the reduced data set of 33 features, samples extracted with each of the different solvents are well separated in the PCA indicating the efficiency of secondary metabolite extraction differs by solvent (Fig. 3a). The same separation of solvents is observed at macroscale (Supplementary Fig. 5; Supplementary Table 2), though the time required to run macroscale experiments with low-volatility solvents was markedly longer (requiring 20-fold longer solvent evaporation times) than in microscale. We also performed PCA on the global metabolite profiles (which contain ∼1,000 features) and found that the separation based on solvent persists at both micro and macroscale (Supplementary Fig. 6).

The impact of solvent choice is even more apparent at the level of single features (Fig. 3c). The loadings plot shows the weights of each normalized feature in calculating principal component (PC) 1 and PC 2; in general, if a feature is in the same quadrant on the loadings plot as the sample is in the PCA plot, it is enriched in that sample. Feature 4, for example, was putatively identified as asperfuranone, a polyketide^33^, and was extracted almost exclusively in γ-caprolactone. This is demonstrated not only by the areas of the extracted peaks (Fig. 3d), but also the fact that the feature falls clearly within the lower left-hand quadrant of the loadings plot, the same quadrant containing all the γ-caprolactone samples in the PCA (Fig. 3b,c). In contrast, features 14–16 were annotated as various adducts of diorcinol, an antibiotic secondary metabolite involved in fungal development^34^, and were collectively extracted best in chloroform (Fig. 3c,e). Feature 28, annotated as a heptaketide^35^, was the only known peak best extracted in pentanol which is why it segregates so strongly on the PCA loadings plot towards the quadrant with the pentanol samples (Fig. 3c,f).

These results demonstrate the power of alternative solvents to more fully extract the fungal secondary metabolome. To the best of our knowledge, γ-caprolactone has not been used as an extraction solvent in previous metabolomics studies, likely because its low volatility makes it a challenging solvent to use with traditional extraction methods. The micrometabolomics platform makes it feasible to use γ-caprolactone and other low-volatility solvents to explore segments of the metabolome that simply are not extracted when using more volatile solvents such as chloroform. The geometry of the platform can be simply modified to work with a range of solvents. For example, for solvents of higher volatility, a deeper solvent channel and the addition of a lid make solvents such as ethyl acetate feasible for extractions up to an hour long (Supplementary Fig. 3B; Supplementary Data 2and 3). The range of possible volatilities and the open nature of the platform, which allows for the deposition of solvent-protective coatings, render the micrometabolomics device compatible with a myriad of solvents.

### Culture size impacts the metabolites produced

In mammalian and bacterial cell culture, confinement in micro and nano-litre volumes of fluid leads to profound changes in soluble factor signalling and corresponding functional changes in cell behaviour^18^. These behavioural effects are typically induced by changes in the concentrations of autocrine and paracrine factors when the cell to culture volume ratio is reduced^16^,^36^. We thus aimed to identify the potential effect of culture geometry (well diameter and depth) on the fungal secondary metabolome. It is known that fungal secondary metabolism is affected by spatial factors, such as proximity to other fungi, which differ depending on culture geometry^3^. However, this effect is not often investigated due to the predominance of standard-sized cultureware, such as petri dishes.

**Figure 4:**
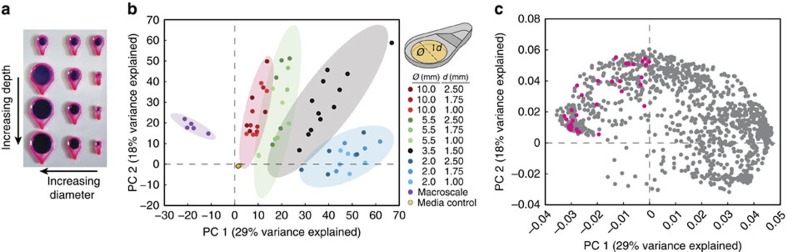
Global metabolite profiles segregate by well diameter but not well depth. (**a**) Panel of devices with varying diameters and depths. (**b**) Principal component analysis of *A. nidulans* grown on GMM agar in wells of varying diameter and depth and extracted with PeOH. Legend values of diameter and depth are given in microns. ‘Macroscale' refers to core samples of fungal growth on a 10-cm petri dish while ‘Media control' refers to core samples of agar alone. Each dot represents one of five independent cultures per condition from one experiment and the shaded ellipses represent 95% confidence intervals for each of the different diameters or control conditions. (**c**) Loadings plot of individual features for the PCA in **b**. Each dot represents one feature. Features in pink were putatively annotated as *A. nidulans* secondary metabolites based on exact mass <10 p.p.m. error (Supplementary Table 3).

We designed a panel of devices with different depths and diameters of the central culture well (Supplementary Fig. 3C; Supplementary Data 4) in which we analysed the landscape of metabolites produced by *A. nidulans* (Fig. 4a). The cultures were inoculated and extracted in proportion to the surface area of the well, which was also used to scale feature intensity after LC-MS analysis. The PCA demonstrates a clear relationship between culture well diameter and global metabolite profile (Fig. 4b). As expected, this trend clusters the largest diameter microwells nearest to the macroscale culture. Interestingly, culture well depth had little impact on the global metabolite profiles. Features coloured pink in the loadings plot (Fig. 4c) correspond to features putatively annotated as *A. nidulans* secondary metabolites by exact mass (Supplementary Table 3). Interestingly, all of these compounds fall in the upper left hand quadrant of the loadings plot. This implies that despite scaling by the surface area of the well, these metabolites are produced to a greater extent in macroscale and large diameter microscale cultures; it is not simply that larger wells have proportionately more compound produced.

The spatial distribution of secondary metabolism throughout a fungal culture may explain the changes observed in different diameter microwells. Some secondary metabolites are made primarily in the spores of the fungus while others are made primarily in the hyphae^22^,^37^,^38^. In a point-inoculated culture, spores are produced in the centre of the colony with hyphae radiating towards the edges^39^. The change in the ratio of culture surface area to culture perimeter between the small and large wells could explain the differences in metabolite composition based on developmental stage of the fungus. There are a number of features primarily found in wells of small diameter that are not annotated as secondary metabolites. Some of these could be the uncharacterized *A. nidulans* secondary metabolites (which are estimated to comprise ∼60% of the *A. nidulans* metabolome) not produced in traditional macroscale culture^31^. The micrometabolomics platform moves beyond the limited geometries offered by standard petri dishes and can be adapted to include more complex shapes that could have a significant impact on secondary metabolite production.

### Culture of *A. fumigatus* on blood

While fungi are typically considered opportunistic pathogens in humans and have not likely experienced evolutionary pressure towards producing metabolites that specifically target the human host, it has been shown that secondary metabolites play an important role during infection^40^,^41^. There are many characterized *A. fumigatus* secondary metabolites implicated in virulence including verruculogen (interacts with the epithelial lining of the respiratory tract^42^), endocrocin (inhibits neutrophil recruitment^40^), fumagillin (causes neutrophil toxicity^43^), hexadehydroastechrome (unknown mechanism^44^) and gliotoxin (induces apoptosis in macrophages and inhibits phagocytosis and ROS production in neutrophils^41^). With the exception of gliotoxin and hexadehydroastechrome, macroscale studies have not been published documenting whether these metabolites can be detected during pathogenesis; there is a gap between compound discovery and compound relevance to disease.

**Figure 5:**
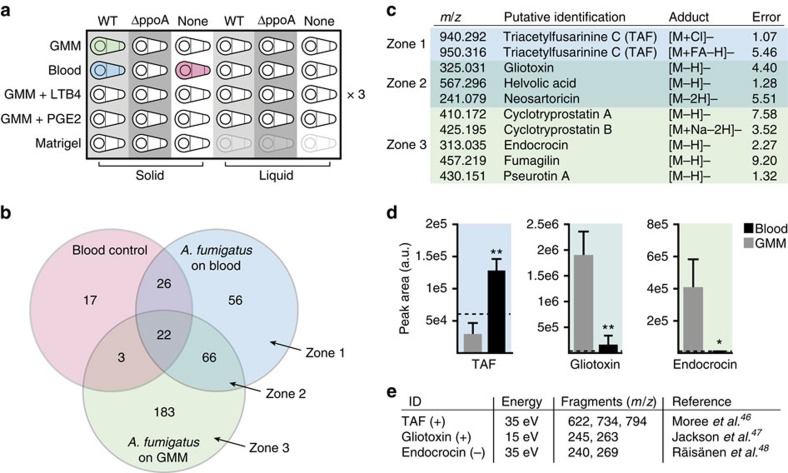
*A. fumigatus* metabolite production varies when grown on blood as compared to GMM. (**a**) Experimental conditions tested in the micrometabolomics platform in triplicate. Data for all three independent cultures from one experiment are shown in **b**–**e**. (**b**) Overlap of global metabolite profiles extracted with PeOH from blood, *A. fumigatus* grown on blood, and *A. fumigatus* grown on GMM. Peaks were extracted and aligned using XCMS online. Numbers represent peaks unique in *m*/*z* value and retention time. (**c**) Putative annotation of peaks based on exact mass of known *A. fumigatus* secondary metabolites. Error given in p.p.m. Adducts are compatible with the observed spectrum and colour corresponds to peak location in **b**. (**d**) Peak areas for three putative secondary metabolites produced by *A. fumigatus* grown on either blood or GMM. The peak areas for both TAF adducts were summed. The dotted line is the peak threshold of 60,000 below which is considered noise. Error bars represent s.d. of three microchannels. *P* values were calculated using unpaired Student's *t*-tests: *P value <0.05, ^**^ *P* value <0.005. (**e**) LC-MS/MS of the three peaks with putative IDs in **d**. Replicates were pooled prior to LC-MS/MS. TAF and gliotoxin were analysed in positive mode, endocrocin in negative mode.

Using the micrometabolomics platform, a large panel of microenvironments can be tested, including rare and expensive matrices that better mimic conditions during disease. We performed an experiment to screen secondary metabolite production across three types of fungal inoculum, five types of fungal media, and two modes of culture all in triplicate (Fig. 5a). *A. fumigatus* was selected as it is the best characterized opportunistic fungal pathogen in the context of secondary metabolism. We chose different culture environments that represent chemical landscapes that can be found in the human lung, including the presence of blood cells and eicosanoids—fatty acid derivatives that are key in immune regulation. Synthetic eicosanoids are expensive, typically costing hundreds of dollars per 100 μg or requiring custom synthesis. The *ΔppoA* mutant has one of the three fungal cyclooxygenase-like enzymes deleted and the mutant with all three cyclooxygenase-like enzymes deleted is hypervirulent in a mouse model^45^. The media conditions supplemented with eicosanoids were selected with the thought that they might differentially impact the *ΔppoA* mutant.

When wild type (WT) *A. fumigatus* grown in solid culture on blood is compared with growth on solid GMM, there are evident changes in its global metabolite profile (Fig. 5b). In particular, zone 1 contains 56 features that are produced primarily when *A. fumigatus* is grown on blood. The 66 features in zone 2 are produced by *A. fumigatus* on both blood and GMM, and the 183 features in zone 3 are produced primarily when *A. fumigatus* is grown on GMM. Features in zones 1–3 were compared with databases of known *A. fumigatus* secondary metabolites and annotated by exact mass (error <10 p.p.m.) (Fig. 5c). Of these putative annotations, one metabolite from each zone was selected for further analysis. The peak areas for each feature support its zone classification, with triacetylfusarinine C produced primarily when the fungus is grown on blood, endocrocin (a spore metabolite) produced primarily when the fungus is on GMM, and gliotoxin produced on either medium (Fig. 5d). The putative IDs of these three features were confirmed by tandem mass spectrometry (LC-MS/MS) (Supplementary Table 4); retention times and fragments of gliotoxin and endocrocin matched purchased standards (retention time difference <30 s; fragment *m*/*z* difference <0.05) and fragments of all three compounds matched the published literature (Fig. 5e)^46^,^47^,^48^.

Features in zone 1 are produced only when *A. fumigatus* is grown on blood. These features are of significant interest as they could be more relevant to fungal virulence than features present solely on GMM agar. The only feature that could be annotated in zone 1 is triacetylfusarinine C (Fig. 5d). The microenvironment of blood induced production of this siderophore (iron binding molecule)^49^, which was otherwise produced only at low levels. The other treatments in this screen yielded few significant differences: the *ΔppoA* mutant was similar to WT and the supplemented GMM conditions were similar to GMM alone. The micrometabolomics platform allowed us to efficiently test culture conditions and going forward, the ease of using this platform will enable experiments with more biologically accurate microenvironments to help uncover secondary metabolites with relevance to disease progression, biomarker diagnosis and therapeutic discovery that would otherwise be hidden.

### Coculture of fungi and bacteria

Interkingdom communication (for example, fungal-bacterial) is prevalent and causes changes in secondary metabolite production as a defence mechanism or in reaction to stress^13^. For example, culture with *B. gladioli* causes *R. microsporus* to produce enacyloxin antibiotics^50^ and the coculture of *Sphingomonas* and *A. fumigatus* isolates leads to the production of glionitrin A^51^. Traditional cocultures are performed either by completely mixing the culture or by inoculating a plate with the two organisms side by side. In a mixed culture, both organisms must be able to grow in the same media and it can be difficult to separate physical effects from soluble factor effects. In a side by side culture, there is significant spatial heterogeneity; microbes in each culture are of differing distance from the other culture. We extended the micrometabolomics platform to allow culture and extraction of two organisms in soluble communication (Supplementary Fig. 3D; Supplementary Data 5) to address these challenges.

**Figure 6:**
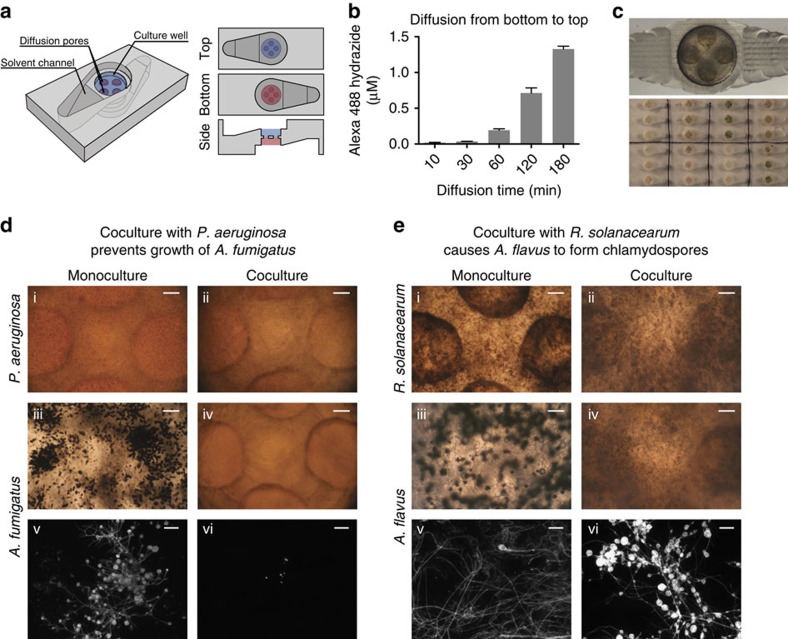
A microscale coculture platform that enables segregated analysis of interkingdom communication. (**a**) The coculture platform is comprised of two micrometabolomics devices in diffusion contact via four pores in the floor of each culture well. (**b**) Diffusion of a 25-μM solution of fluorescent dye, Alexa 488 hydrazide, through the agar culture pads into the upper solvent well is time dependent. Error bars represent s.d. of three devices. (**c**) Photos of bacterial and fungal growth within the coculture devices. (**d**) Monoculture and coculture of *P. aeruginosa* and *A. fumigatus* after 3 days at 30 °C shows that coculture prevents *A. fumigatus* growth. (**e**) Monoculture and coculture of *R. solanacearum* and *A. flavus* for 3 days at 30 °C shows that coculture causes *A. flavus* to generate chlamydospores. Images were taken at × 4 ((**d**,**e**) i–iv), scale bar, 250 μm, and a subset of wells were stained with calcafluor white and imaged at × 10 ((**d**,**e**) v–vi), scale bar, 25 μm. Images are representative of three culture wells.

The coculture device is made up of two micrometabolomics platforms placed on each opposing face of a thermoplastic layer and connected via pores in the bottom of each culture well (Fig. 6a). This allows for diffusion of secreted chemical factors between the two cultures while keeping the bulk of each culture separate. The cultures are both exposed to air, have a nearly uniform distance from each other, and can be grown on different media in the same device. To characterize the contact between the two culture wells, diffusion of a fluorescent dye, Alexa 488, across the two agar pads was measured over time. Over 3 h, there is clear movement of the fluorophore from the bottom compartment where it was applied, through the agar, to the top compartment (Fig. 6b). This indicates that the coculture device allows compounds to diffuse between the cultures on a biologically relevant timescale and can be extracted on each side of the platform.

To test the feasibility of using the coculture device to study interkingdom interactions, we cultured fungi together with bacteria, all of which were able to grow successfully in the devices (Fig. 6c). Besides monoculture of each organism, the human pathogen *Pseudomonas aeruginosa* was cocultured with *Aspergillus fumigatus*, and plant pathogen *Ralstonia solanacearum* (*R. solanacearum*) was cocultured with *Aspergillus flavus* (*A. flavus*) and *Fusarium sporotrichioides*, two common plant pathogenic fungi. Despite growing well in monoculture, *A. fumigatus* is unable to grow when in coculture with *P. aeruginosa* (Fig. 6d). In addition, *A. flavus* has a dramatic induction of chlamydospore formation when in coculture with *R. solanacearum* (Fig. 6e) as does *F. sporotrichioides* to a lesser extent (Supplementary Fig. 7). Chlamydospores are large, thick-walled cells that are induced by environmental stressors including bacterial metabolites^52^. The coculture platform enables simple phenotypic screening of multikingdom cocultures while the integrated solvent extraction channels make it possible to do chemical analysis of secreted metabolites in the two cultures. Taken together, the coculture extension of the micrometabolomics device opens up another important microenvironmental factor, signals from surrounding organisms, to ready manipulation.

## Conclusions

We introduce a microscale culture platform to study microbial secondary metabolism in response to engineered chemical, mechanical and geometrical microenvironmental cues as well as coculture with other organisms. Our open microculture platform is compatible with liquid media or solid agar fungal culture methods, and provides the opportunity to incorporate mammalian or microbial cell types in coculture. Furthermore, the device is easily arrayed and can be used to screen microenvironmental factors that may have an effect on secondary metabolite production, including incorporation of biological samples such as blood, sputum, mucus, extracellular matrix components, etc. The microscale culture well makes it feasible to do large studies with rare samples, such as biological fluids or cells from asthma patients who experience exacerbations from inhaling fungal spores.

In addition, we developed an integrated, open, passive, biphasic metabolite extraction system that leverages surface tension forces to operate at microscale. The device is compatible with a wide range of solvents, giving users access to previously uninterrogated segments of the metabolome. The extraction process can be multiplexed and automated, providing increased efficiency for large parameter studies, while still providing sufficient recovery of metabolites to perform LC-MS. The platform has multiple strengths, including the identification of candidate metabolites and their required culture conditions prior to larger scale structural elucidation studies, the appearance or disappearance of peaks of interest in rare or biologically relevant matrices, or the identification of metabolite differences related to observable physiological changes of the microorganism. Our results demonstrate that the micrometabolomics platform can be used to investigate how the fungal microenvironment influences metabolite production and set the stage to use the platform as a fast and simple way to probe secondary metabolites hidden within cryptic gene clusters.

## Methods

### Device fabrication

Devices were micromilled as previously described^53^. Briefly, polystyrene sheets of 3 mm thickness were purchased from Goodfellow (#ST313300). Features were cut out of the polystyrene using a CNC micromilling machine (Tormach pCNC 700). Devices used for testing different solvents were coated with Parylene C (CAS #28804-46-8) using a Specialty Coating Systems PDS 2010 vacuum deposition system; 25 g Parylene C dimer was used to get a 15-μm thick coating.

### Coculture device characterization

Diffusion in the coculture device (Fig. 6b) was assessed by filling the culture compartments with a 7% low melt agarose gel. Alexafluor 488 hydrazide (Invitrogen) was diluted to 25 μM in PBS and applied to the solvent well of the bottom compartment. The device was turned over and PBS was added to the top solvent well. Periodically, the PBS in the top well was removed for fluorescence analysis using a BMG Pherastar Multimode plate reader with excitation at 485 nm and emission at 520 nm.

### Fungal culture

Strains of *A. nidulans* (WT, RDIT 9.32)^54^ and *A. fumigatus* (WT, Af293; *ΔppoA*, TDWC 1.13)^55^ were maintained as glycerol stocks. They were activated by culture on solid GMM^32^ made with low-gelling temperature agar (CAS #39346-81-1) for 4 days at 37 °C at which point conidia were collected in 0.01% Tween-80.

For control macroscale cultures, 200 μl spores at a concentration of 10e5 spores per ml were spread over the surface of GMM in a 10-cm petri dish. For microscale cultures, the culture well was filled with 20 μl media and 4 μl spores were spread at a concentration of 10e4 spores per ml over the top. Blood agar was made using 96% human blood (Bioreclamation LLC) with 4% low-gelling temperature agar or PBS for solid and liquid cultures, respectively. Matrigel media was made with 50% Basement Membrane Matrix (BD biosciences) and 50% GMM without agar. Where indicated, conditions supplemented with eicosanoids were made by adding 1 μM LTB_4_ or PGE_2_ (Cayman Chemical) to the spores prior to inoculation. *A. nidulans* cultures were grown at 30 °C and *A. fumigatus* cultures were grown at 37 °C for 3–4 days in a humidified chamber to prevent evaporation.

### Fungal and bacterial coculture

*P. aeruginosa* (PA 14, from Dr Yun Wang, Northwestern University) and *A. fumigatus* (Af293, from CBS, Centraalbureau voor Schimmelcultures Fungal Biodiversity Centre of the Royal Netherlands Academy of Arts and Sciences) in both mono- and coculture were grown on potato dextrose agar (PDB from BD, Franklin Lakes, NJ, mixed with low melt agar). *R. solanacearum* (GMI1000, from ATCC, Manassas, VA), *A. flavus* (NRRL 3357, from CBS) and *F. sporotrichioides* (from University of Wisconsin-Madison, Department of Plant Pathology Teaching Lab) were all grown in mono- or coculture on ISP2 media^56^. *P. aeruginosa* and *R. solanacearum* cultures were inoculated with 2 μl overlay of 10e8 bacteria per ml. *A. fumigatus* and *A. flavus* cultures were inoculated with 2 μl overlay of 10e6 spores per ml. *F. sporotrichioides* was inoculated with a punch of macroscale culture; a wide orifice pipet tip was used to transfer consistent amounts of fungal culture from macroscale to the microscale devices. Monoculture studies were done by inoculating the same organism in both the top and bottom culture wells. Cultures were grown at 30 °C for 3 days in a humidified chamber to prevent evaporation.

In coculture experiments, fungal cell walls were stained (Fig. 6d,e v, vi) by adding 10 μl calcofluor white (1 mg ml^−1^) to the wells 1 min prior to microscopy. Cultures were collected from coculture devices by cutting the edges of the agar pads using a sterile 18 g needle and aspirating into 250 μl large orifice pipet tips (USA Scientific). Cultures were wet mounted using another 10 μl ddH_2_O and imaging was done using a Zeiss Axio Imager A10 microscope (Carl Zeiss, Oberkochen, Germany) equipped with a Zeiss A Plan- × 10 lens, a series 120 X-Cite light source (EXFO), and a DAPI excitation/emission filter set. Images were collected with AxioVision Release 4.7 software (Carl Zeiss).

### Metabolite extraction

Metabolites in the macroscale samples were extracted by removing 10 cores of 10 mm diameter from the culture and homogenizing them in 0.01% Tween-20. The homogenized suspension was removed to a glass vial and a volume of solvent, 2.449 ml, proportional to the culture surface area of the cores was added. Samples were agitated every 5 min for 30 min after which they were centrifuged at 2,500 r.p.m. (608*g*) for 10 min. The solvent layer was removed to a fresh vial for evaporation as below. Metabolites were extracted from the microscale cultures by pipetting solvent into the tapered end of the teardrop channel. Solvent volumes used were proportional to the surface area of the culture; for standard 3.5 mm diameter microscale cultures, 30 μl solvent was used. The metabolites were allowed to passively diffuse into the solvent layer for 30 min after which the solvent was collected into glass vials. Samples were evaporated to dryness (1–4 h) in a vacuum concentrator (Thermo Express SC250EXP) without heat, except samples extracted with γ-caprolactone that were heated to 37 °C for the first hour of evaporation.

### Metabolite analysis

Extracts were re-dissolved in 50 μl of 20% DMSO/MeOH and centrifuged at 10,000*g* for 10 min. Sample order was randomized and a 10μl portion was examined by high performance liquid chromatography with diode-array detection (HPLC-DAD) and MS or MS/MS analysis. HPLC-DAD-MS was done using an Agilent 6210 TOF LC-MS. The same reverse-phase C18 column (Alltech Prevail C18 2.1 mm by 100 mm with a 3-μm particle size) was used for all samples at a flow rate of 125 μl per min. The solvent gradient was 95% acetonitrile (MeCN)/H_2_O (solvent B) in 5% MeCN/H_2_O (solvent A), both containing 0.05% formic acid, as follows: 0% solvent B from 0 to 5 min, 0 to 100% solvent B from 5 to 35 min, 100% solvent B from 35 to 40 min, 100 to 0% solvent B from 40 to 45 min and reequilibration with 0% solvent B from 45 to 50 min. Nitrogen was used as auxiliary and sheath gas. Source voltage was set at 4,000 V, nebulizer pressure at 20 psig, drying gas flow rate at 10 l per min and drying gas temperature at 350 °C.

HPLC-MS/MS was done using a ThermoFinnigan LCQ Advantage ion trap mass spectrometer. The solvent gradient was as described above for HPLC-MS. Source heater temperature was set to 0 °C, sheath flow rate was set to 49.22, current was set to 5.52 μA, voltage was set to 5.03 kV, capillary temperature was set to 275 °C and capillary voltage was set to −15.74 V. The MS/MS fragmentation scheme, including collision energies, for confirmation of three putatively identified compounds (Fig. 5) is described in Supplementary Table 4.

### XCMS analysis

Agilent.d files were converted to.mzXML files using ProteoWizard^57^. They were then grouped by condition and uploaded to XCMS online^58^,^59^,^60^. Multigroup analyses were performed using default HPLC/ UHD Q-TOF settings that have been optimized for an HPLC run with ∼60 min gradient and subsequent analysis using a high-resolution ESI-QTOF-MS. Polarity was specified to be negative. The XCMS multigroup analysis includes retention time correction, peak picking using the centWave algorithm, and peak grouping algorithms.

### Peak identification and PCA

Data on each of the peaks was downloaded and imported into MATLAB. Peaks were removed from analysis if they had a *q* value, as calculated by XCMS, of <0.05 or were present in fewer than 66% of replicates of at least one condition in the experiment. Peaks were also removed if present in the solvent control or if their maximum intensity was <5,000 (60,000 for all peaks in the matrix experiment because of higher background in blood). Putative identification of peaks was made if the observed *m*/*z* matched the predicted *m*/*z* within 10 p.p.m. error when checking against the Reaxys database (version 2.20770.1, Elsevier Information Systems GmbH, Frankfurt, Germany) limited to secondary metabolites of *A. nidulans* or *A. fumigatus* depending on the experiment and an in-house database of secondary metabolites for these two fungal species. Putative identifications were discarded if proposed adducts were incompatible with those present in the spectrum. For adducts annotated as the same compound, retention times agreed within 45 s. Each peak was scaled to have unit variance and additional scaling by surface area was performed for experiments using devices with varying diameters. PCA was done using the PCA function in MATLAB with the singular value decomposition algorithm.

### Statistical analysis of metabolite levels

Statistical analysis was performed using GraphPad Prism 6 software. To compare metabolite extraction efficiency with different solvents (Fig. 3d–f), we used the non-parametric Kruskal–Wallis test with Dunn's multiple comparison correction. To compare metabolite levels between two different growth conditions, Student's *t*-test was used (Fig. 5d).

## Additional information

**Accession codes:** Processed mass spectra were deposited in the XCMS repository (https://xcmsonline.scripps.edu/) with the following accession codes: Data set ID 1023001 (data associated with Fig. 3), Data set ID 1026465 (data associated with Fig. 4) and Data set ID 1036638 (data associated with Fig. 5). The mass spectra have undergone retention time alignment and peak picking algorithms as well as basic statistical comparisons, but no further post processing has been done to the stored data.

**How to cite this article:** Barkal, L. J. *et al.* Microbial metabolomics in open microscale platforms. *Nat. Commun.* 7:10610 doi: 10.1038/ncomms10610 (2016).

## Supplementary Material

Supplementary Information Supplementary Figures 1-7, Supplementary Tables 1-4 and Supplementary References

Supplementary Movie 1 Operation of the micrometabolomics device. In the movie, 1-pentanol is pipetted over the surface of PBS containing dye and is then recovered without disturbing the PBS interface.

Supplementary Data 1 Microscale culture extractor CAD file.

Supplementary Data 2 High solvent volume extractor CAD file.

Supplementary Data 3 High solvent volume lid CAD file.

Supplementary Data 4 Multisize culture extractors CAD file.

Supplementary Data 5 Coculture extractor CAD file.

## Figures and Tables

**Table 1:** Putative annotations of peaks isolated from *A. nidulans* culture on GMM agar subsequently extracted with chloroform, γ-caprolactone or pentanol.

**ID**	**Annotation**	**m/z**	**Adduct**	**Error**
1	Cordycepin	501.194	[2M−H]−	3.88
2	Cordycepin	286.070	[M+Cl]−	5.88
3	Arugosin G	537.255	[M+FA−H]−	9.73
4	Asperfuranone	377.157	[M+FA−H]−	8.43
5	Aspoquinolone A/B	502.165	[M+K−2H]−	2.27
6	Austinol intermediate (C25H30O7)*	441.192	[M−H]−	0.49
7	Dehydroaustinol	493.131	[M+K−2H]−	7.81
8	Dehydroaustinol	501.177	[M+FA−H]−	1.67
9	Dehydroaustinol	491.149	[M+Cl]−	1.76
10	Dehydroaustinol	455.171	[M−H]−	0.63
11	Dehydrocitreoisocoumarin or 2-acetoacetyl T4HN	137.024	[M−2H]−	1.60
12	Desacetylaustin or austinol	457.187	[M−H]−	1.22
13	Desacetylaustin or austinol	493.164	[M+Cl]−	0.65
14	Diorcinol	229.085	[M−H]−	6.70
15	Diorcinol	459.178	[2M−H]−	6.26
16	Diorcinol	505.182	[2M+FA−H]−	9.59
17	Emericellamide A	646.354	[M+K−2H]−	6.66
18	Emericellamide A	644.380	[M+Cl]−	0.05
19	Emericellamide C/D	640.393	[M+FA−H]−	0.66
20	Emericellamide C/D	630.365	[M+Cl]−	0.96
21	Emericellamide C/D	632.339	[M+K−2H]−	6.08
22	Emericellamide E/F	658.395	[M+Cl]−	0.17
23	Emericellamide E/F	668.425	[M+FA−H]−	1.34
24	Emericellamide E/F	660.375	[M+K−2H]−	0.60
25	Emericellin	393.172	[M−H]−	2.16
26	Emericellin	815.386	[2M−H]−	7.26
27	Emodic acid	336.977	[M+K−2H]−	2.86
28	A heptaketide (C15H24O2)†	257.154	[M+Na−2H]−	7.21
29	Isoaustinone	471.202	[M+FA−H]−	1.88
30	Isoaustinone	463.153	[M+K−2H]−	0.39
31	Nidulalin A or B	301.072	[M−H]−	0.73
32	Nidulol	239.057	[M+FA−H]−	2.41
33	Variecoxanthone A	679.254	[2M−H]−	1.74

Compound ID numbers match Fig. 3c. *m*/*z* is mass to charge ratio detected by the mass spectrometer. Annotations were made by comparing observed masses with databases of known *A. nidulans* secondary metabolites. Annotations were only made if the error (difference between predicted and measured mass) was <10 p.p.m. Predicted adducts are compatible with the observed spectra and adducts annotated as the same compound eluted within 45 s of each other.

^*^Based on exact mass, the austinol intermediate could be neoaustinone, austinolide or 11β-hydroxyisoaustinone.

^†^Based on exact mass, the heptaketide formula could take multiple structures.
