# Supplementary material for: Microbial metabolomics in open microscale platforms
Source: Nat Commun. 2016 Feb 4;7:10610. doi: 10.1038/ncomms10610 (PMC4742997; doi:10.1038/ncomms10610)
Supplement: Supplementary Information — Supplementary Figures 1-7, Supplementary Tables 1-4 and Supplementary References [file ncomms10610-s1.pdf]

Supplementary Figures

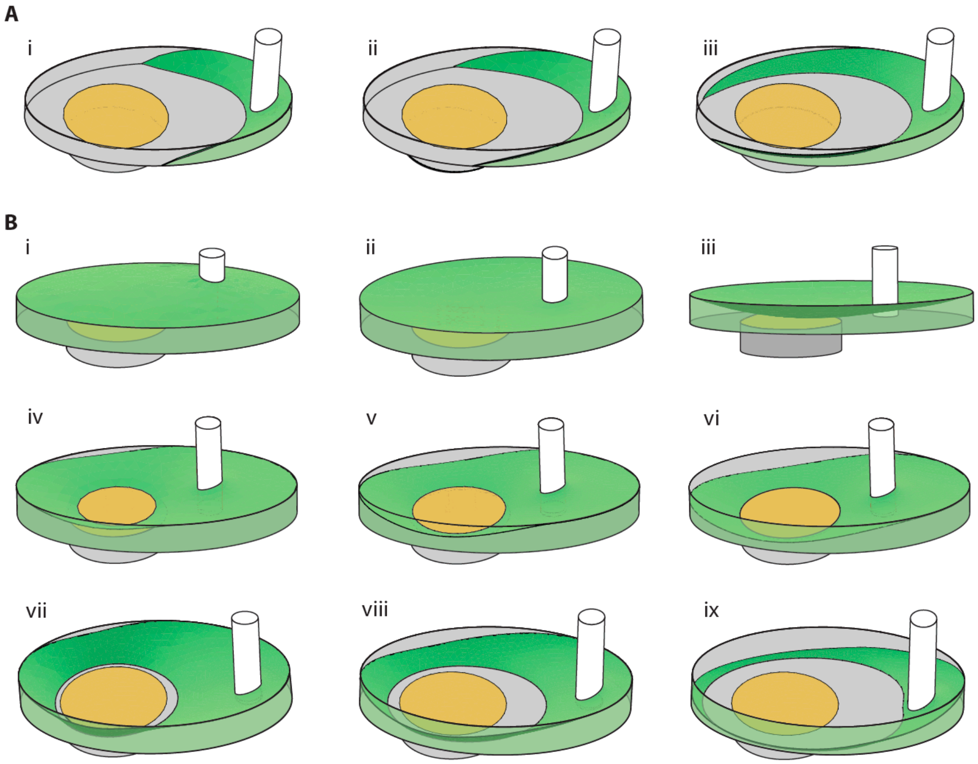

Supplementary Figure 1. Simulation of solvent flow extended from Figure 1. (A) Filling of the device demonstrates thin filaments of solvent around the edges of the device. (B) Metabolite recovery proceeds when solvent (green) is removed from the micrometabolomics device through the pipette tip (white). Notably, aqueous media (yellow) forms a dome that touches the surface of the solvent (B. iii), but remains intact throughout the simulation.

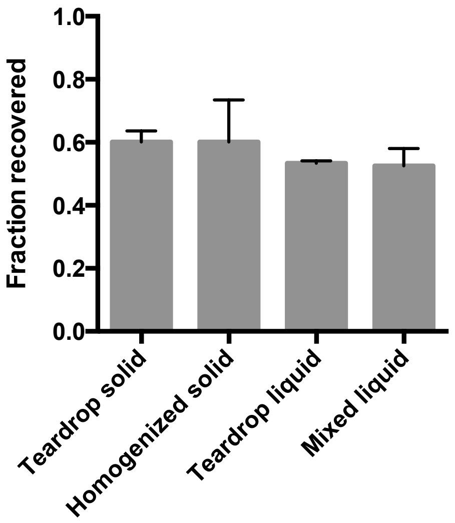

Supplementary Figure 2. Microscale extraction efficiency is comparable to traditional methods. Gliotoxin standard was mixed into solid GMM or liquid RPMI and extracted with PeOH for 30 minutes using the teardrop platform or traditional gel homogenization (solid agar sample) or vortexing (liquid media sample). Extracts were evaporated completely and resuspended in 20% ACN + 1% FA prior to measurement by HPLC-UV/Vis. Error bars represent standard deviation of three technical replicates.

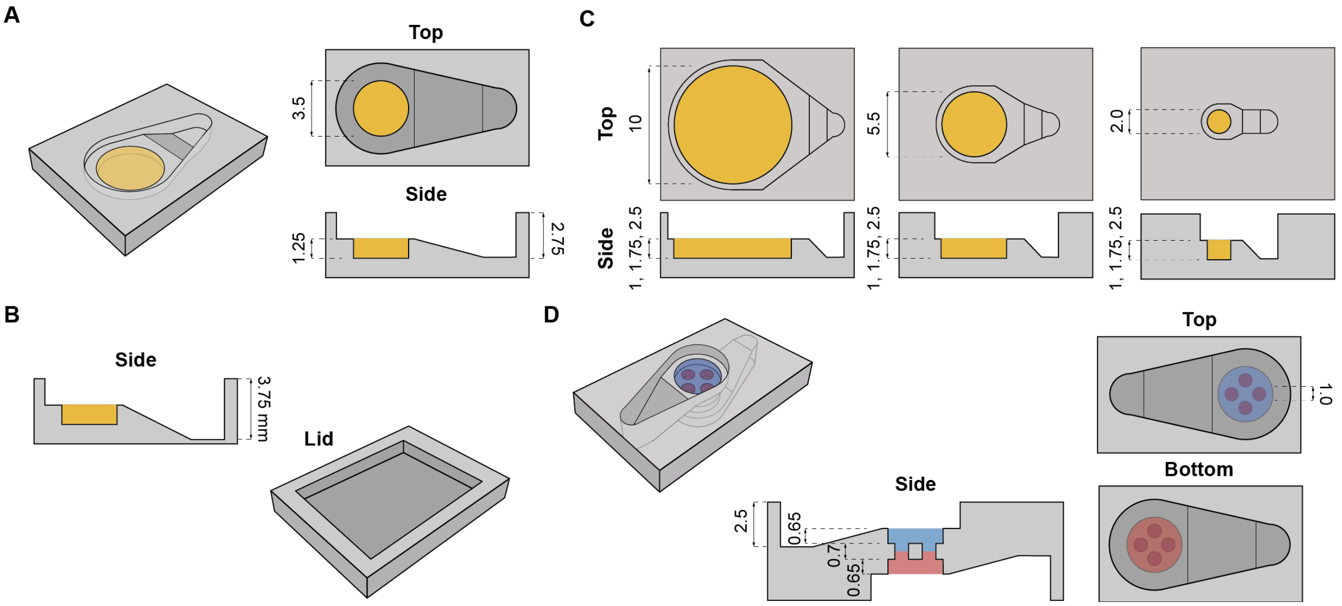

Supplementary Figure 3. Device schematics. (A) Single culture extractor used to collect the data presented in Figures 3, 5, and Supplementary figures 1, 2, 4, 5, 6. (B) High solvent volume extractor and lid. These devices are compatible with more volatile solvents (e.g., ethyl acetate) as they can accommodate greater volumes of solvent, up to 70 μL. Used with the extractor lid, this device allows ethyl acetate to be used for extractions up to 1 h long before evaporation prevents solvent recovery. (C) Multisize culture extractors with three different diameters and depths that were used to collect the data presented in Figure 4. (D) Coculture device with integrated extraction that was used to collect the data presented in Figure 7. All measurements are given in mm.

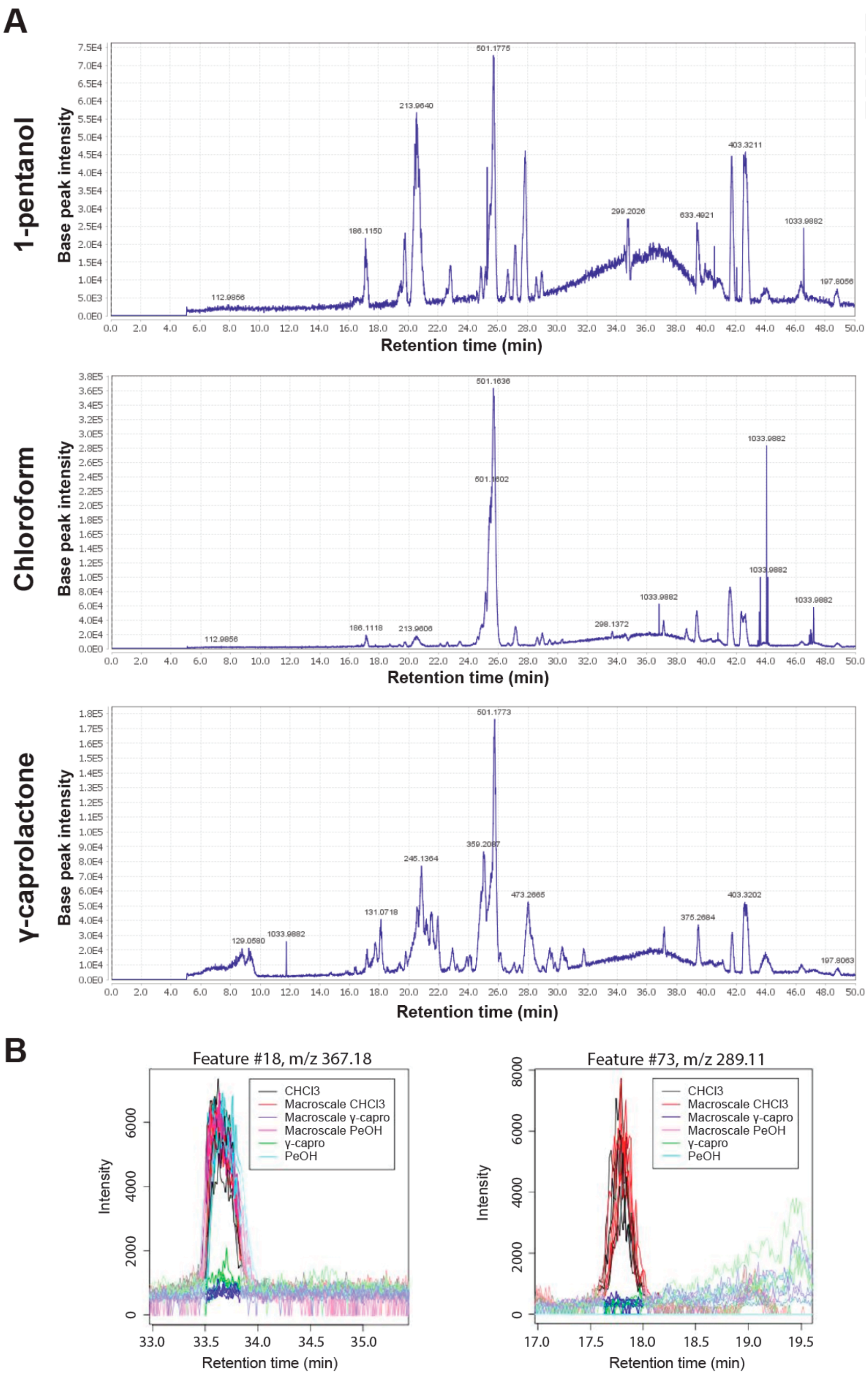

Supplementary Figure 4. Picking peaks from chromatograms. (A) Base peak chromatograms for representative microscale *A. nidulans* cultures extracted with 1-pentanol, chloroform, and γ-caprolactone demonstrate gross differences in metabolites. Retention time is in minutes. (B) Examples of relatively low intensity peaks picked in XCMS that meet criteria for inclusion in later analyses (Methods, Peak identification).

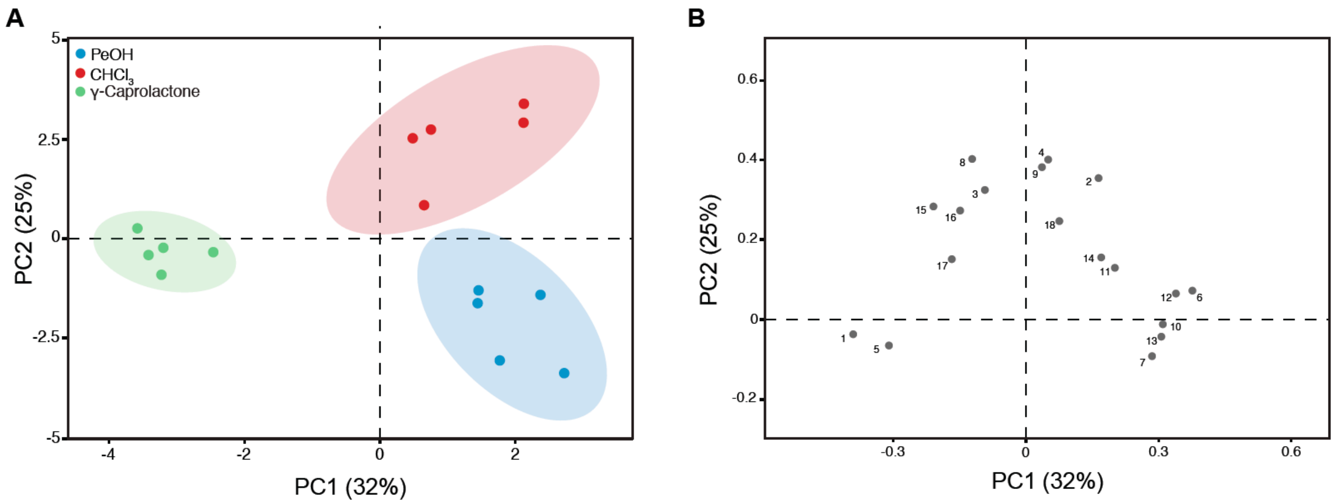

Supplementary Figure 5. Solvents extract different secondary metabolite profiles from macroscale cultures. (A) Principal component analysis of *A. nidulans* cultured at macroscale, in traditional 10 cm plates. Only features that could be annotated as known secondary metabolites (listed in Supplementary Table 2) were used in the PCA. Each dot represents one of five independent cultures per condition from one experiment and the shaded ellipses represent 95% confidence intervals. (B) Loadings plot for the PCA in (A) in which each dot represents a feature used in the PCA.

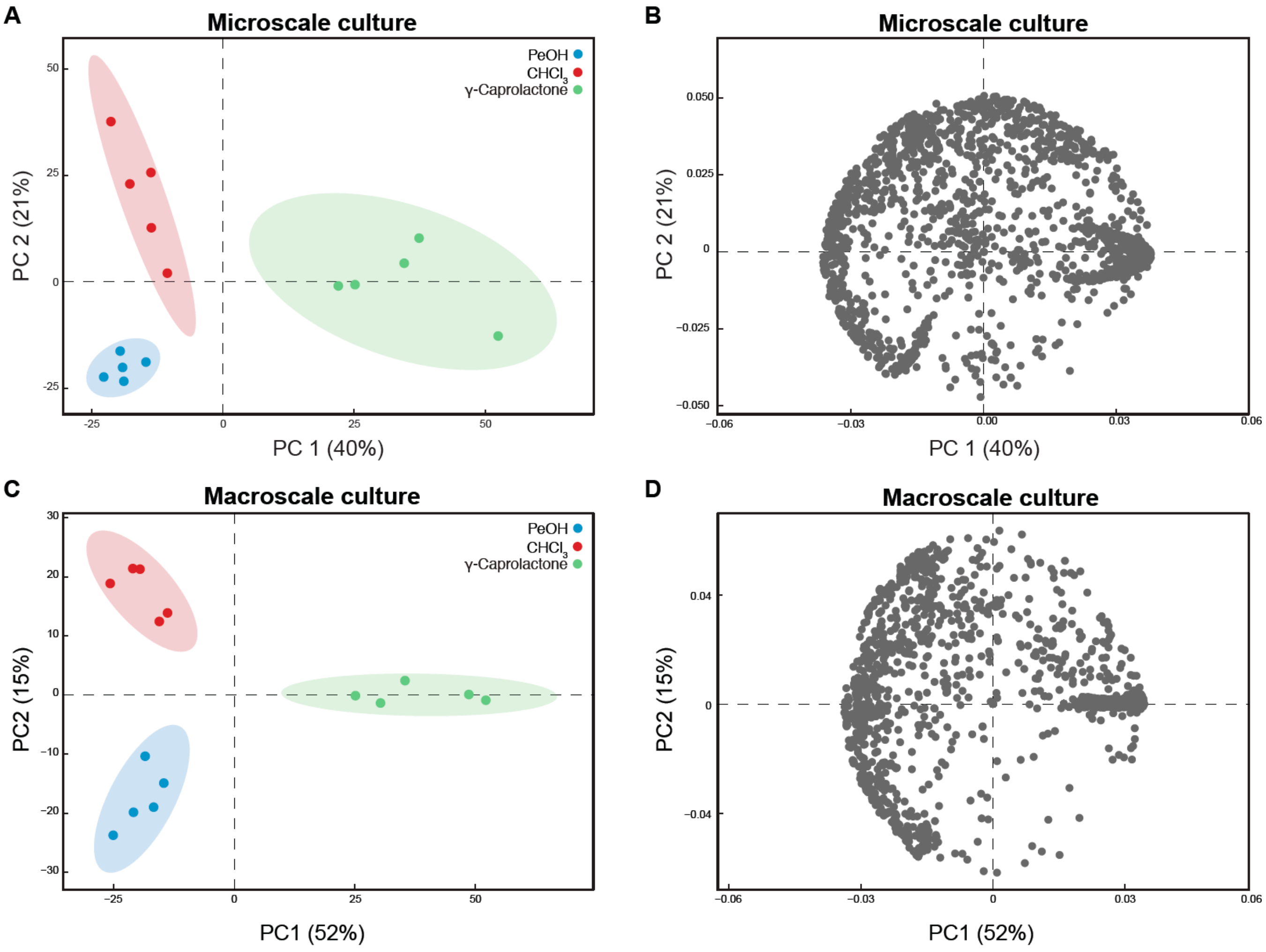

Supplementary Figure 6. Solvents extract different global metabolite profiles from both microscale and macroscale cultures. (A, C) Principal component analysis of global metabolite profiles of *A. nidulans* cultured in the micrometabolomics platform (A) or macroscale culture (C). Each dot represents one of five independent cultures per condition from one experiment and the shaded ellipses represent 95% confidence intervals. (B, D) Loadings plots for the PCAs in A and C, respectively, in which each dot represents a feature used in the PCA.

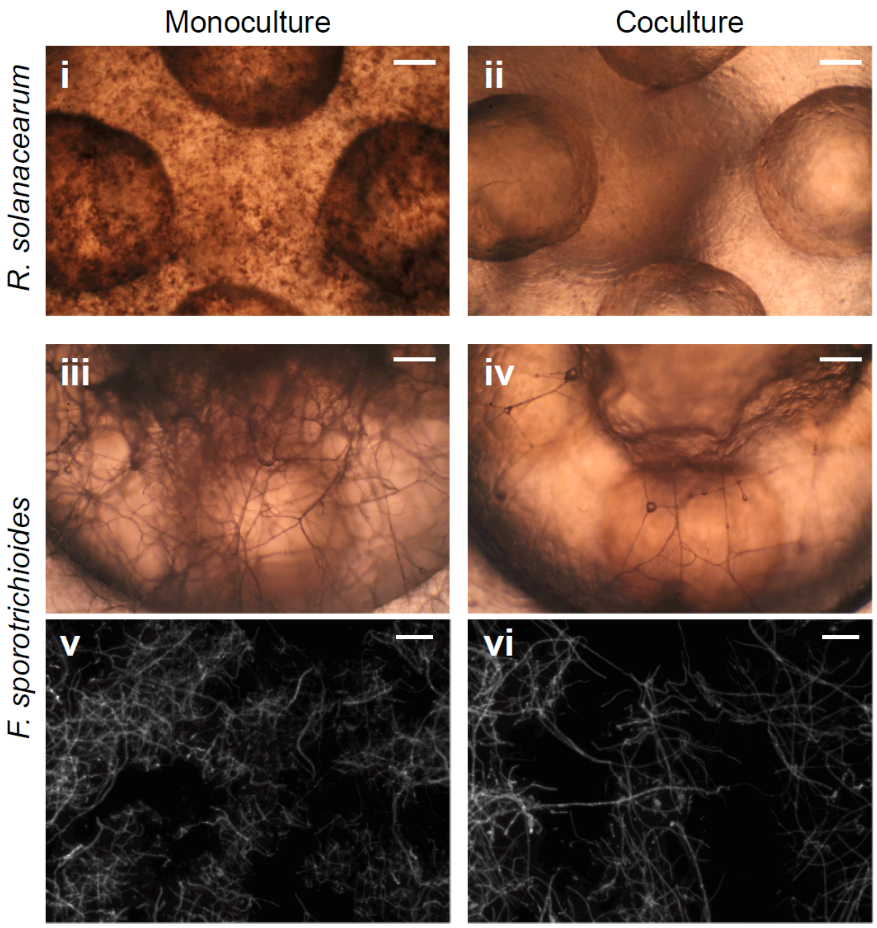

Supplementary Figure 7. Coculture with *R. solanacearum* causes *F. sporotrichioides* to produce chlamydospores. *R. solanacearum* and *F. sporotrichioides* were cultured separately or in diffusion contact for 3 days at 30°C. Images were taken at 4X (i-iv, scale bars = 250 μm) and a subset of wells were stained with calcofluor white and imaged at 10X (v-vi, scale bars = 25 μm). Images are representative of three culture wells.

Supplementary Tables

Supplementary Table 1. Physical properties of solvents used to extract *A. nidulans* cultures described in Figure 3. Boiling point values are from ChemSpider and log P values are predicted using ACD/Labs Percepta Platform - PhysChem module.

Solvent	Chemical Formula	Boiling Point (°C)	LogP
**Chloroform**	$CHCl_3$	61	1.50
**1-Pentanol**	$C_5H_{12}O$	137	1.41
**γ-Caprolactone**	$C_6H_{10}O_2$	219	0.26

Supplementary Table 2. Putative annotations of peaks isolated from macroscale *A. nidulans* culture in 10cm plates on GMM agar subsequently extracted with chloroform, γ-caprolactone, or pentanol as described in Figure 3. Annotations were made by exact mass compared with databases of known *A. nidulans* secondary metabolites. Compound ID numbers match Supplementary Fig. 5B. Adducts are compatible with the observed spectra, adducts annotated as the same compound eluted within 45 seconds of each other, and mass error is given in ppm.

ID	Annotation	m/z	Adduct	Error
**1**	Asperfuranone	377.161	[M+FA-H]-	2.94
**2**	Austinoneol	459.202	[M+FA-H]-	5.59
**3**	Austinoneol	449.174	[M+Cl]-	2.52
**4**	Austinoneol	459.202	[M+FA-H]-	4.10
**5**	Dehydroaustinol	491.148	[M+Cl]-	9.61
**6**	Dehydrocitreoisocoumarin or 2-acetoacetyl T4HN	137.024	[M-2H]-	1.01
**7**	Dihydromonacolin L	341.189	[M+Cl]-	7.78
**8**	Diorcinol	229.087	[M-H]-	5.48
**9**	Diorcinol	229.087	[M-H]-	1.85
**10**	Emericellin	393.171	[M-H]-	3.83
**11**	Emericellin	393.171	[M-H]-	1.62
**12**	Emindole DA	426.278	[M+Na-2H]-	2.00
**13**	Heptaketide	235.170	[M-H]-	1.61
**14**	Monacolin N	325.179	[M+Na-2H]-	5.47
**15**	N-acetyl-6-hydroxy-DL-tryptophan	261.088	[M-H]-	8.30
**16**	N-acetyl-6-hydroxy-DL-tryptophan	261.088	[M-H]-	7.78
**17**	o-Orsellinic acid	503.120	[3M-H]-	7.27
**18**	preaustinoid A3, A4, or A5	455.208	[M-H]-	2.82

Supplementary Table 3. Putative annotations of peaks isolated from *A. nidulans* culture on GMM agar in microscale wells of varying depth and diameter subsequently extracted with 1-pentanol as described in Figure 4. Annotations were made by exact mass compared with databases of known *A. nidulans* secondary metabolites. Adducts are compatible with the observed spectra, adducts annotated as the same compound eluted within 45 seconds of each other, and mass error is given in ppm. **Based on exact mass, the austinol intermediate could be neoaustinone, austinolide, or 11β-hydroxyisoaustinone.

ID	Annotation	m/z	Adduct	Error
1	1(3H)-isobenzofuranon, 3-(2,6-dihydroxyphenyl)-4-hydroxy-6-methyl	271.061	[M-H]-	0.43
2	1(3H)-isobenzofuranon, 3-(2,6-dihydroxyphenyl)-4-hydroxy-6-methyl	543.130	[2M-H]-	0.65
3	3,5-dimethylorsellinic acid	151.076	[M-H-CO2]-	1.34
4	3,5-dimethylorsellinic acid	195.066	[M-H]-	0.67
5	Aniduquinolone A	480.198	[M+FA-H]-	9.25
6	Aniduquinolone B	498.213	[M+FA-H]-	1.10
7	Alternariol	257.047	[M-H]-	3.93
8	Asperthecin	339.013	[M+Na-2H]-	3.47
9	Austinol intermediate (C25H30O7)**	441.192	[M-H]-	0.11
10	Austinol intermediate (C25H30O7)**	509.179	[M-H+NaCOOH]-	1.44
11	Austinol intermediate (C25H30O7)**	477.169	[M+Cl]-	0.44
12	Austinol intermediate (C25H30O7)**	883.391	[2M-H]-	0.68
13	Austinol intermediate (C25H30O7)**	905.373	[2M-2H+Na]-	0.88
14	Austinoneol	873.403	[2M+FA-H]-	3.57
15	Citreoisocourmarin	311.052	[M+Na-2H]-	5.55
16	Dehydroaustinol	441.155	[M-H-CH2]-	0.96
17	Dehydroaustinol	455.168	[M-H]-	6.94
18	Dehydrocitreoisocoumarin	137.025	[M-2H]-	1.05
19	Desacetylaustin or austinol	915.382	[2M-H]-	0.79
20	F-9775A/B	395.075	[M-H]-	5.85
21	Isoaustinone	425.194	[M-H]-	7.20
22	Isoaustinone	471.198	[M+FA-H]-	9.17
23	Isoaustinone	851.401	[2M-H]-	0.31
24	Monodictyphenone	287.056	[M-H]-	2.15
25	Monodictyphenone	243.067	[M-H-CO2]-	1.53
26	Norsolorinic acid anthrone	177.055	[M-2H]-	1.98
27	Norsolorinic acid anthrone	401.123	[M+FA-H]-	2.05
28	Orsellinic acid	167.034	[M-H]-	5.72
29	Protoaustinoid A	467.218	[M+K-2H]-	6.33
30	PsiAβ	295.226	[M-H]-	7.06
31	PsiAβ	613.442	[2M-2H+Na]-	5.64
32	Terrequinone A	535.223	[M+FA-H]-	1.82
33	Variecoxanthone A	679.261	[2M-H]-	9.06

Supplementary Table 4. MS/MS fragmentation scheme for confirmation of three putatively identified compounds is described in Figure 5. Ions with the target m/z (a) detected within the retention time (RT) window (b) were selected for fragmentation with the indicated collision energies (c). While there is no commercially available TAF standard, gliotoxin and endocrocin standards were fragmented (d) to compare with the experimentally observed fragments (e). Observed fragments were also compared to the published literature (f).

Target m/z[a]	Putative ID	Lower RT bound[b]	Upper RT bound[b]	Collision energy[c]	Standard fragments[d]	Observed fragments[e]	Ref.[f]
**906.3**	TAF [M+H]+	17.9 min	19.4 min	35 eV	*not available	794, 734, 622	Moree et al.[1]
**327.0**	Gliotoxin [M+H]-	20.7 min	22.2 min	15 eV	245, 263	245, 263	Jackson et al.[2]
**313.0**	Endocrocin [M-H]-	23.9 min	25.4 min	35 eV	225, 269	225, 269	Räisänen et al.[3]

Supplementary References

1. Moree, W. J. *et al.* Interkingdom metabolic transformations captured by microbial imaging mass spectrometry. *Proc. Natl. Acad. Sci. U.S.A.* **109,** 13811–13816 (2012).
2. Jackson, L. C., Kudupoje, M. B. & Yiannikouris, A. Simultaneous multiple mycotoxin quantification in feed samples using three isotopically labeled internal standards applied for isotopic dilution and data normalization through ultra-performance liquid chromatography/electrospray ionization tandem mass spectrometry. *Rapid Commun. Mass Spectrom.* **26,** 2697–2713 (2012).
3. Räisänen, R., Björk, H. & Hynninen, P. H. Two-dimensional TLC separation and mass spectrometric identification of anthraquinones isolated from the fungus *Dermocybe sanguinea*. *Z. Naturforsch C.* **55,** 195–202 (2000).
